# *CASP4* gene silencing in epithelial cancer cells leads to impairment of cell migration, cell-matrix adhesion and tissue invasion

**DOI:** 10.1038/s41598-018-35792-8

**Published:** 2018-12-07

**Authors:** Giuliana Papoff, Dario Presutti, Cristiana Lalli, Giulia Bolasco, Simonetta Santini, Candida Manelfi, Valentina Fustaino, Stefano Alemà, Giovina Ruberti

**Affiliations:** 1 National Research Council, Institute of Cell Biology and Neurobiology - Campus Adriano Buzzati-Traverso Via E. Ramarini, 32 00015 Monterotondo (Rome), Italy; 2 Epigenetics and Neurobiology Unit, European Molecular Biology Laboratory (EMBL), Rome, Via E. Ramarini 32 Monterotondo (Rome), Italy

## Abstract

Inflammatory caspases, including human caspase-4 (CASP4), play key roles in innate immune responses to promote fusion of phagosomes harboring pathogenic bacteria with lysosomes, halt intracellular replication of pathogens, maturation and secretion of pro-inflammatory cytokines. The role of inflammatory caspases in cancer cells remains poorly investigated. Here, we explored the consequences of modulating *CASP4* expression levels on the migratory behavior of epithelial cancer cell lines. By a gene silencing approach and *in vitro* and *in vivo* studies we show that down-regulation of CASP4 leads to impaired cell migration and cell-matrix adhesion. This phenotype is accompanied by an increased actin cytoskeleton polymerization, changes in the overall organization of adherens junctions (AJs) and number and size of focal adhesions. Interestingly, the cell migration deficit could be reversed by epithelial growth factor treatment, and depletion of calcium ions unveiled a role of CASP4 in the novo assembly of AJs, suggesting that the role of CASP4 is not cell-autonomous. Finally, *CASP4*-silenced A431 cells exhibited a severe reduction in their ability to invade lung tissue, when injected into nude mice. Overall, our data support the emerging evidence that inflammatory caspases can regulate cell migration through actin remodeling and uncover a novel role of CASP4 in cancer cell behavior.

## Introduction

During bacterial infection, different sensing systems cooperate to detect and control infections also through the assembly of inflammasomes, high-molecular weight cellular complexes, with recruitment and activation of inflammatory caspases^1^. This in turn promotes the maturation and secretion of the pro-inflammatory interleukin (IL)-1β and IL-18 cytokines for controlling inflammatory responses and pyroptotic cell death^2,3^. Inflammatory caspases namely human and mouse caspase-1 (CASP1), human caspase-4 (CASP4) and caspase-5 (CASP5) and mouse caspase-11 (CASP11), are cysteine-aspartic proteases characterized by the presence of a caspase recruitment domain (CARD) at the N-terminus. Both CASP11 and CASP4/5 bind intracellular lipopolysaccharide (LPS), the major structural element of Gram-negative bacteria outer membrane, with high specificity and affinity through their N-terminal CARD, leading to caspase activation and pyropoptotic death^4^. CASP4 has also been involved in endoplasmic reticulum (ER) stress-induced apoptosis^5,6^ a conclusion challenged by other studies^7^ and as a positive regulator of TNF-α induced NF-kB signaling^8^.

Interestingly, CASP11 can regulate multiple events, during an inflammatory response, including actin dynamics and cell migration. Indeed, *CASP11*−/− leukocytes are defective in migration *in vitro* and *in vivo* and CASP11 appears to regulate actin depolymerization through the interaction with the actin interacting protein 1 (Aip1), an activator of cofilin-mediated actin depolymerization, independently of its enzymatic activity^9^. Moreover, it has been shown that CASP11 and CASP4/5 promote the fusion of phagosomes, harboring pathogenic bacteria, with lysosomes by modulating actin polymerization^10,11^.

The expression of *CASP11* though highly inducible upon LPS injection and stress is barely detectable in most tissues of healthy mice, whereas *CASP4* is highly constitutively expressed in normal placental and lung tissues and in several cancer cell lines suggesting that it can play other functions besides its involvement in innate immune responses^12–14^.

To metastasize successfully, cancer cells have to detach from their original location, to migrate, invade a blood or lymphatic vessel, travel in the circulation to a distant site and establish a new cellular colony. Detachment, migration, invasion are inter-related essential metastatic steps affected by complex biochemical events. Cell migration involves the integration of signals that define cell polarity, dynamic remodeling of cytoskeleton and focal adhesion structures as well as the regulation of the adhesive interaction with the extracellular environment. Tumor microenvironment in which cells interact with each other and with the extracellular matrix, extracellular growth factors and cytokines play significant role in cancer initiation and progression.

Here, we report that downregulation of CASP4 modifies the behavior of human cancer epithelial cell lines by decreasing their cell detachment, cell migration, cell invasion features and increasing actin polymerization and the number and size of focal adhesions. Moreover, *CASP4-*silencing impacts cell-cell and cell-matrix interaction under exogenous stimuli (i.e. EGF and calcium). Finally, *CASP4*-silenced A431 cells exhibit a strikingly reduced invasive behavior upon dissemination to the lung of *in vivo* injected mice.

## Results

### *CASP4*-silencing in human epithelial cancer cell lines impairs cell migration

**Figure 1:**
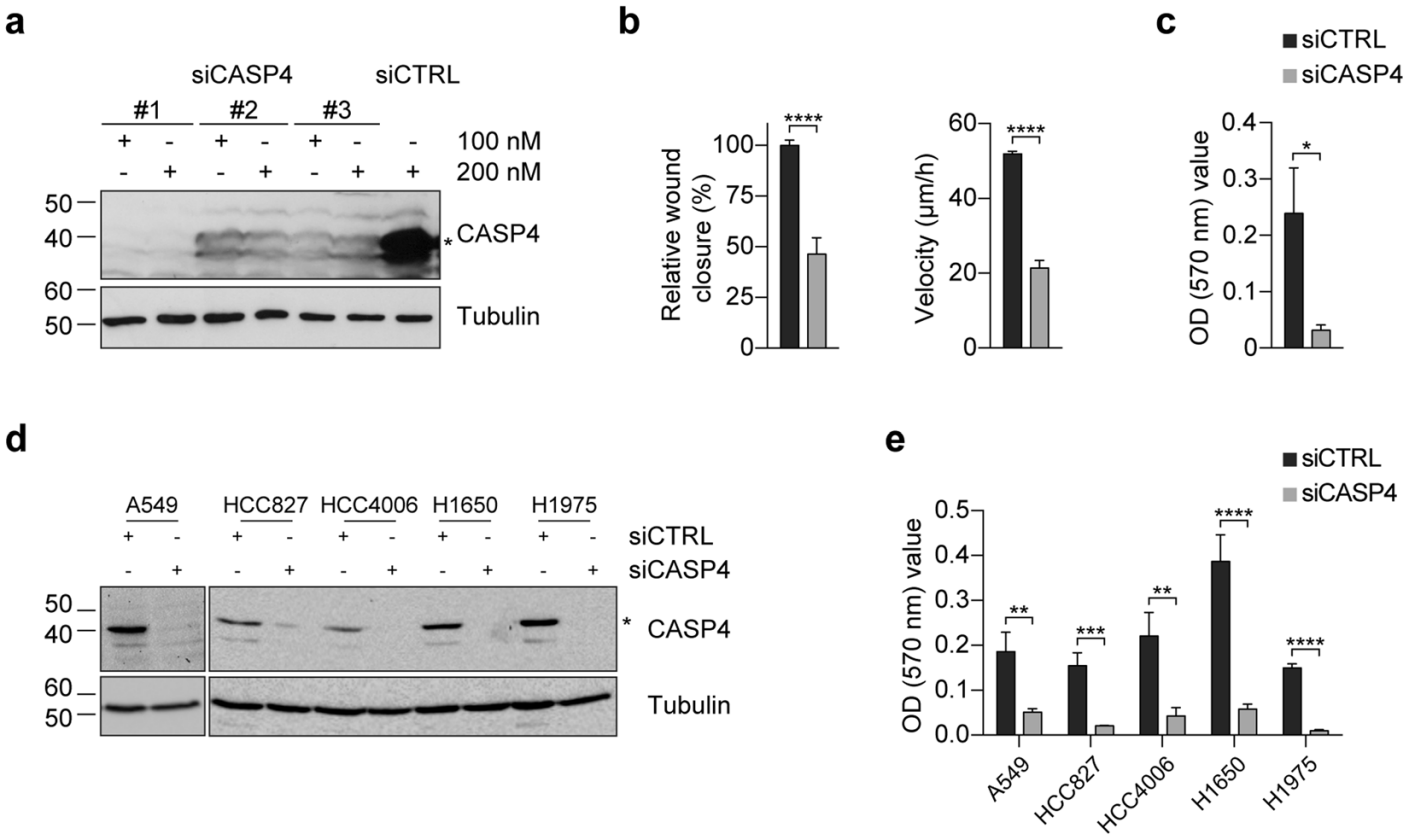
*CASP4*-silencing in epithelial cancer cell lines inhibits cell migration. (**a**) Western blot analysis of CASP4 and tubulin expression in A431 cells transfected with the indicated siRNA (100–200 nM). siCTRL was luciferase siRNA in all experiments. (**b**) Analysis of A431 cell migration by wound healing assays. The wound closure was quantified at 8 hours post-wound by measuring the cell-free area using the ImageJ software^35^. Bar plots represent the percentage of relative wound closure (on the left) and migration velocity (on the right), calculated as described in materials and methods. Data were obtained by the analysis of 11 microscopy images and they are representative of two independent experiments (relative wound closure: p < 0.0001, n = 11; migration velocity: p < 0.0001, n = 44 corresponding to 4 length values/image). (**c**) Analysis of A431 transwell cell migration assays. Bar plots represent the optical reading (OD_570 nm_) of FBS-guided cell migration of siCTRL and siCASP4 transfected cell lines (p = 0.01, n = 5). The data are representative of two independent experiments. (**d**) Western blot analysis of CASP4 and tubulin expression in NSCLC cells transfected with siCTRL and siCASP4 #1 at 100 nM concentration. (**e**) Analysis of NSCLC transwell cell migration assays. Bar plots represent the optical reading (OD_570 nm_) values of FBS-guided cell migration of siCTRL and siCASP4 #1 transfected cell lines allowed to migrate for 15–20 hours (A549: p = 0.008, n = 5; HCC827: p = 0.0004, n = 9; HCC4006: p = 0.003, n = 11; H1650 and H1975: p < 0.0001, n = 11 and n = 14). The data are representative of three independent experiments. Statistical analysis was performed by Wilcoxon rank sum test for the comparisons of siCASP4 with the siCTRL transfected cell lines. Significant p-values are represented by asterisks: *p < 0.05; **p < 0.01; ***p < 0.001; ****p < 0.0001. Non-significant p-values are not shown.

In order to investigate the effects of CASP4 modulation on cell growth, apoptosis and cell migration, cancer cell lines were transfected with small interfering RNA (siRNA) targeting *CASP4*. All siRNA targeting exons coding for the pro-domain (#3) or the large p20 (#2) or the small p10 (#1) subunits strongly decrease CASP4 expression in A431 cells (Fig. 1a). The siRNA #1 also silenced very efficiently *CASP4* in several epithelial lung cancer cell lines (Fig. 1d). Whereas *CASP4-*silencing with siRNA #1 had no effect on A431 cell viability or apoptosis (Supplementary Fig. S1b,c), it strongly impacted cell migration of A431 cells (Fig. 1b,c and Supplementary Fig. S1a). Two separate methods were used to investigate cell migration: wound healing assay and transwell migration assay. *CASP4*-silencing strongly reduced the percentage of relative wound closure (54% reduction) and of migratory speed (0.6-fold reduction) in A431 cells (Fig. 1b). Importantly, *CASP4*-silencing also significantly decreased transwell migration in A431 cells (Fig. 1c) and the other cancer cell lines (Fig. 1e). To minimize the possibility of RNAi off-target effects, the wound healing assays were also performed with siRNA #2 and #3 and obtained similar results to siCASP4 #1. In particular the assays showed a reduction in the percentage of relative wound closure of 75% and 33% respectively (Supplementary Fig. S1a), indicating that impairment of cell migration indeed resulted from *CASP4*-silencing.

**Figure 2:**
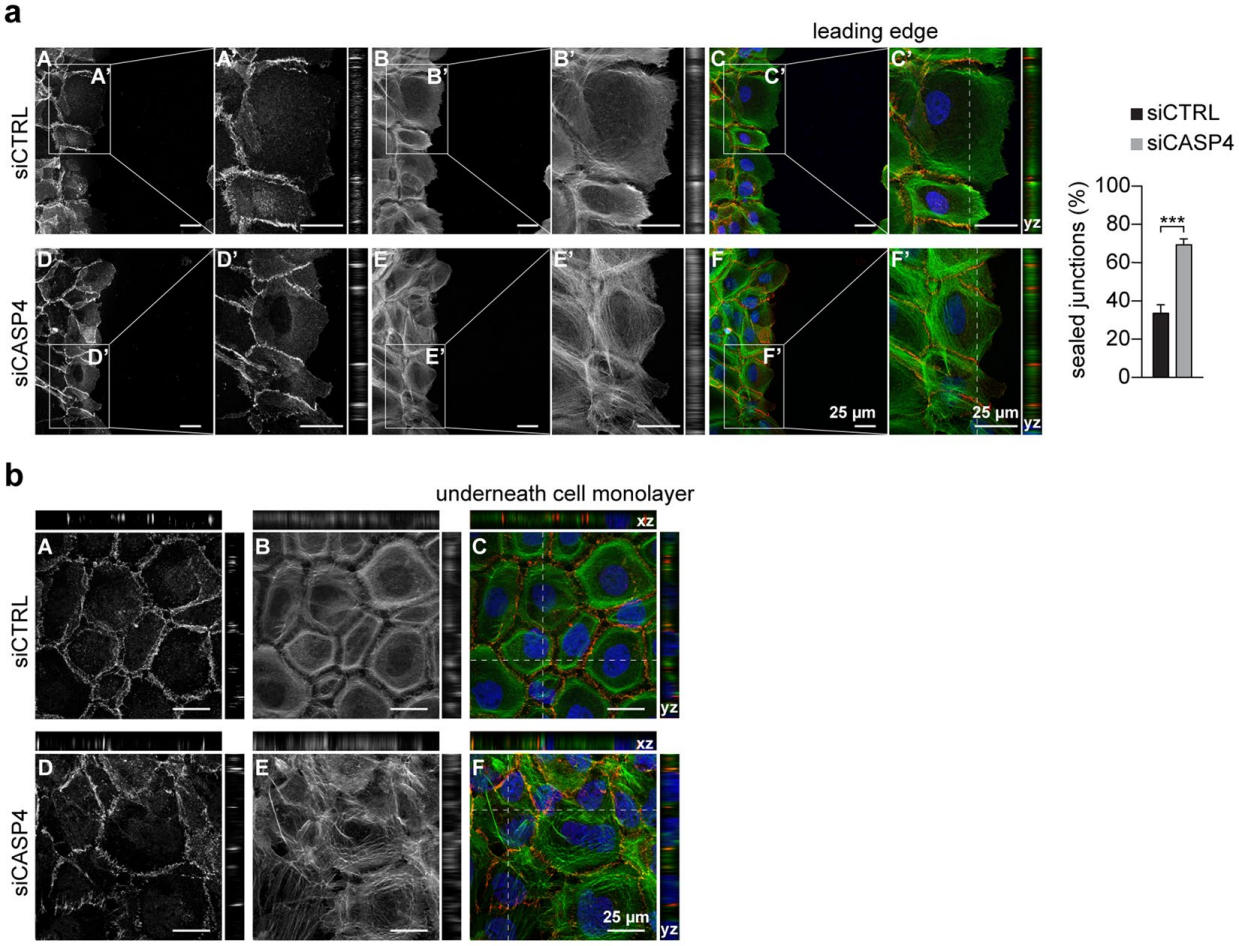
Effects of *CASP4*-silencing on cell-cell junctions and actin cytoskeleton polymerization. Representative confocal microscopy images of A431 cells transfected with siCTRL (A–C) or siCASP4 (D–F) stained with E-cadherin reactive antibody (red) (A,D) and phalloidin (green) (B,E) at the leading edge (**a**) and in the underneath confluent cell monolayer (**b**); merged pictures (C,F) are shown. Optical 2x zoom (A’–F’) were analyzed with the *yz* plane projections. Bar plots indicate the percentage of fully sealed junctions at leading edge (p = 0.0003, n = 10). E-cadherin positive junctions were analyzed in 10 confocal microscopy images recorded in two independent experiments; approximately 500 junctions were counted by using ImageJ. In panel (b) both *xz* and *yz* planes are shown. Scale bars (25 µm) are indicated. Statistical analysis was performed by Wilcoxon rank sum test for the comparison of siCASP4 with the siCTRL transfected A431 cells. Significant p-values are represented by asterisks: ***p < 0.001.

Remarkably, at the leading edge of the wound healing, an increase (1 fold) in the number of E-cadherin positive fully sealed cell-cell junctions was detected in *CASP4*-silenced A431 cells (Fig. 2a), consistently with their defective cell migration behavior. Moreover, an increased actin cytoskeleton polymerization, with a large number of stress fibers, was present in *CASP4*-silenced A431 cells particularly in the underneath confluent cell monolayer (Fig. 2b).

We did not observe differences in the expression levels of E-cadherin in control and *CASP4*-silenced A431 cells; moreover both cell lines were negative by immunoblot analysis for N-cadherin and Vimentin, markers associated with mesenchymal phenotypes and increased cell migration ability (Supplementary Fig. S2).

**Figure 3:**
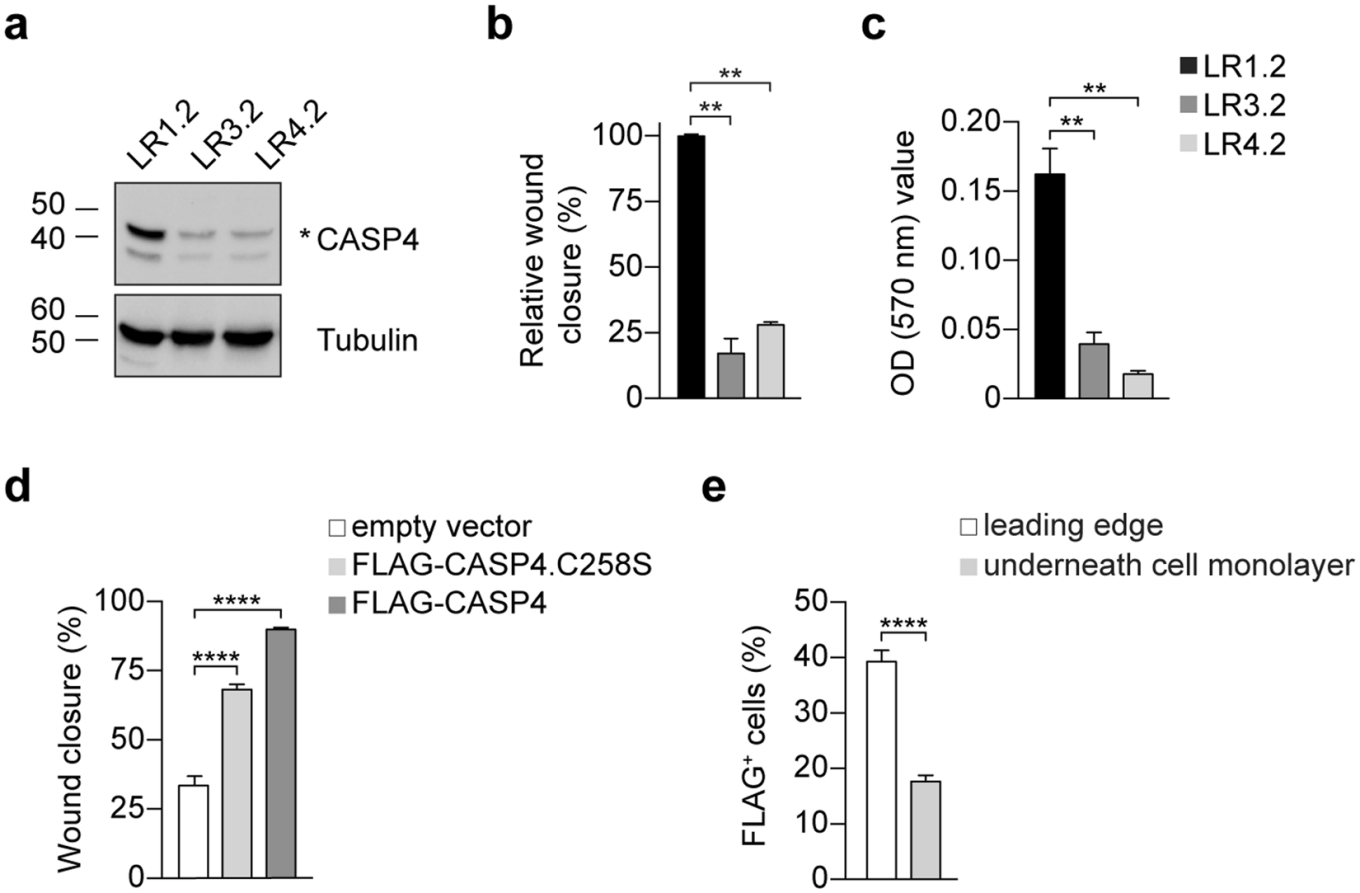
Characterization of stable silenced A431-derived LR cell lines. (**a**) Western blot analysis of CASP4 and tubulin expression in LR cell lines. (**b**,**c**) Cell migration analysis: (**b**) wound closure was quantified in 4–6 images for the indicated cell lines at 8–12 h post-wound. Bar plots represent the percentage of relative wound closure (LR1.2 - LR3.2: p = 0.002, n = 6; LR1.2 - LR4.2: p = 0.009, n = 4); (**c**) transwell cell migration analysis. Bar plots represent the optical reading (OD_570 nm_) of FBS-guided cell migration (LR1.2 - LR3.2: p = 0.002, n = 6; LR1.2 - LR4.2: p = 0.005, n = 6). Data are representative of three independent experiments. (**d**) Analysis of cell migration by wound healing assays in retroviral infected-cells. The wound closure was quantified in 12 images for the indicated cell lines at 6 hours post-wound. Bar plots indicate the percentage of wound closure in the infected LR1.2 cells (FLAG-CASP4.C258S and FLAG-CASP4, p < 0.0001, n = 12). Data are representative of three independent experiments. (**e**) Bar plots indicate the percentage of FLAG^+^ cells in FLAG-CASP4 infected LR1.2 cell line counted in 13 different fields in two independent experiments by using ImageJ (p < 0.0001, n = 13). Statistical analysis was performed by Wilcoxon rank sum test for the comparison of LR3.2 and LR4.2 with LR1.2 cell lines, and with t-test for the comparison of FLAG-CASP4 with control cells, and for the comparisons of percentage of the FLAG^+^ cells. Significant p-values are represented by asterisks: **p < 0.01, ****p < 0.0001. Non-significant p-values are not shown.

To further investigate the effects of *CASP4*-silencing on cell phenotype *in vitro* and *in vivo*, two stable *CASP4*-silenced A431 cell lines - LR3.2 (miRNA CASP4_Hmi402233) and LR4.2 (miRNA CASP4_Hmi402234) with very low or undetectable levels of CASP4 and a control LR1.2 cell line (miR-neg, scrambled) were generated by a miR RNAi lentiviral expression system (Fig. 3a).

As in siRNA transiently transfected A431 cells (Fig. 1), LR3.2 and LR4.2 cells exhibit prominent defects in cell migration, as assessed by wound healing assay (Fig. 3b) and transwell migration assay (Fig. 3c). In particular, a remarkable reduction in the percentage of relative wound closure (LR3.2: 83%, LR4.2: 72%) and transwell cell migration (LR3.2: 0.8-fold reduction; LR4.2: 0.9-fold reduction) was observed. Cell migration defects were also documented in time-lapse experiments by recording the wound healing at the spinning disk (Supplementary Movie 1).

Unfortunately, the rescue of *CASP4* expression in LR3.2 and LR4.2 cells failed because the integrated miRNAs can target any exogenous *CASP4* cDNA. However, LR1.2 control cell line was successfully infected with retroviral expression vectors coding for FLAG-tagged CASP4 or CASP4.C258S, mutated in the protease active site. Approximately 35–60% of LR1.2 cells were infected with the retroviruses (Supplementary Fig. S3a) and both the wild type and the mutated FLAG-CASP4 positively modulated cell migration, indicating that enzymatic activation is not required. A highly significant increase (FLAG-CASP4: 168%, FLAG-CASP4.C258S: 103%) in the wound closure further supports the role of CASP4 in cell migration (Fig. 3d). Interestingly, FLAG^+^ cells were more prone to cell migration and were concentrated at the leading edge (40%, leading edge versus 18%, underneath confluent cell monolayer) (Fig. 3e and Supplementary Fig. S3b).

### E-cadherin distribution in *CASP4*-silenced cells is highly interdigitated at the cell-cell junctions and is modulated by EGF

**Figure 4:**
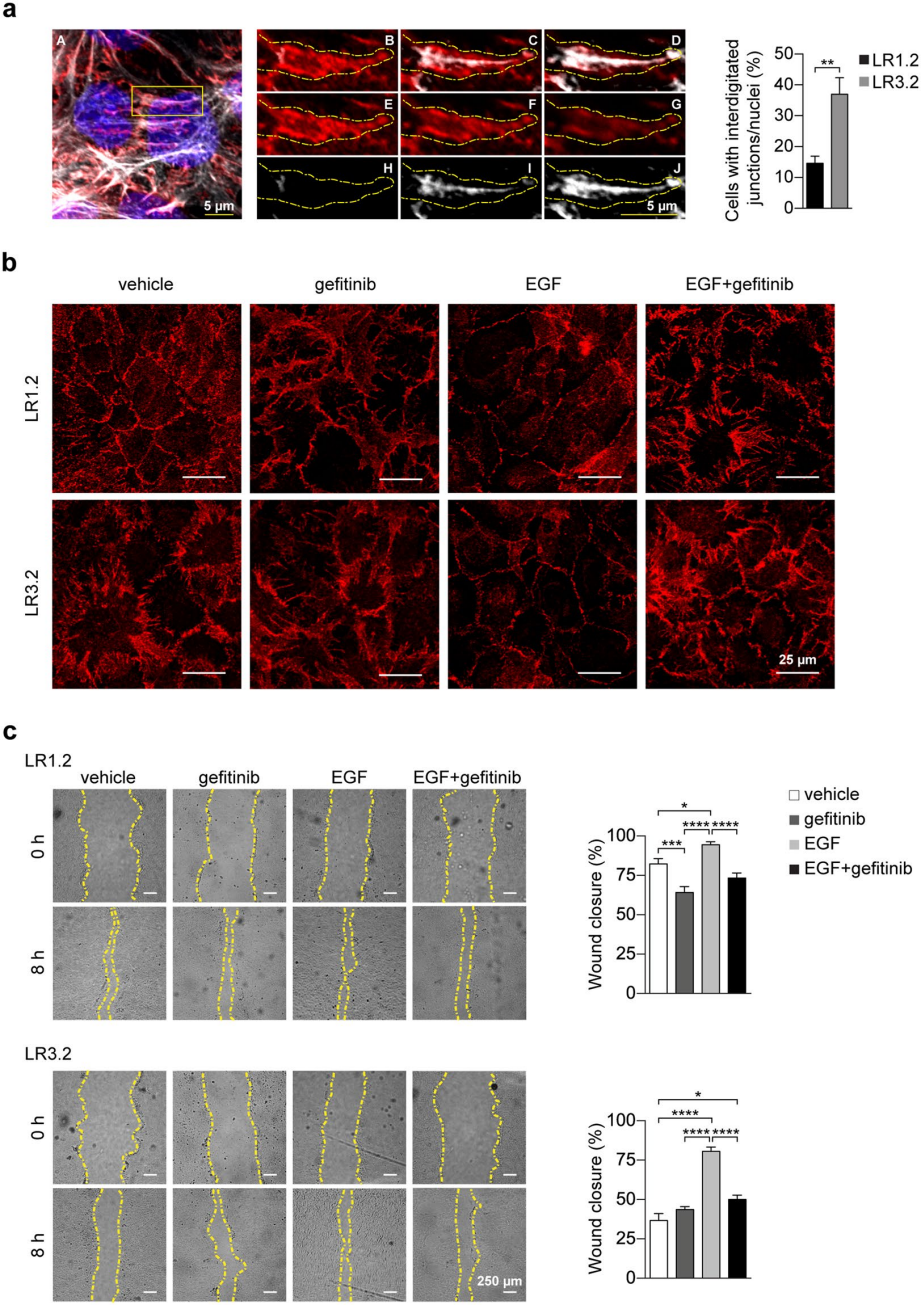
E-cadherin distribution at the cell-cell junctions and its modulation by EGF. (**a**) Representative confocal microscopy projection image of LR3.2 cell line stained with E-cadherin antibody (red), phalloidin-647 (gray) and DAPI (blue). Digital enlargements of the indicated yellow box (A) of three different z planes (B–D; E–G; H–J) of *xy* projections show a finger detail, in merged (B–D), red (E-cadherin) (E-G) and white (phalloidin) (H–J) channels. Scale bars (5 μm) are indicated. Interdigitated E-cadherin junctions were counted in 6 different confocal microcopy images for each cell line in two independent experiments: 1000 cells were counted by using ImageJ. Bar plots indicate the percentage of cells with interdigitated E-cadherin positive junctions respect to the total number of nuclei (p = 0.002, n = 6). Statistical analysis was performed by Wilcoxon rank sum test for the comparison of LR3.2 with LR1.2 cell lines. (**b**) Representative confocal microscopy images of LR1.2 and LR3.2 cell lines stained with E-cadherin antibody (red). Cells were treated as indicated with vehicle (DMSO), gefitinib (300 nM), EGF (50 ng/ml) and EGF + gefitinib for 48 hours in serum free media. Scale bars = 25 µm. (**c**) Representative images of wound healing experiments performed in LR1.2 and LR3.2 cell lines treated with vehicle (DMSO), gefitinib, EGF and EGF + gefitinib. Wounded areas are located within the yellow dashed lines. Scale bars = 250 µm. The wound closure was quantified in 14–16 images for the indicated cell lines at 8 hours post-wound. Bar plots represent the percentage of wound area closure in three independent experiments (LR1.2: gefitinib - DMSO, p = 0.0007; EGF - DMSO, p = 0.04; gefitinib - EGF and EGF - EGF + gefitinib, p < 0.0001. LR3.2: EGF - DMSO, p < 0.0001; EGF + gefitinib – DMSO, p = 0.01; gefitinib - EGF and EGF - EGF + gefitinib, p < 0.0001. n = 14–16). Statistical analysis was performed by ANOVA followed by the Tukey’s post-hoc test for every possible pair comparison. Significant p-values are represented by asterisks: *p < 0.05; **p < 0.01; ***p < 0.001; ****p < 0.0001. Non-significant p-values are not shown.

Untreated and serum-starved *CASP4*-silenced confluent LR3.2 cells show a larger proportion of interdigitated E-cadherin^+^ cell-cell junctions than LR1.2 cells (37% versus 15%) (Fig. 4a), in agreement with the observed cell migration defects. Interdigitated cell-cell junctions, previously described in mammary-derived MCF10A cells are characterized by the presence of actin cables projected from tips of the finger protrusions^15^. Confocal microscopy images of three adjacent z planes of a characteristic finger are shown (Fig. 4a). Interestingly, it has been reported that Epithelial Growth Factor Receptor (EGFR) signaling suppresses this interdigitation and increases mammary-derived MCF10A cell migration^15^. Therefore, AJs behavior was investigated in LR cells treated with EGF or EGF plus gefitinib, a specific EGFR tyrosine-kinase inhibitor^16^_._ Vehicle and gefitinib treatments alone were used as controls. Indeed, in LR3.2 cells treated with EGF, E-cadherin exhibited an uniform plasma membrane staining with smooth boundaries between neighboring cells, whereas the addition of gefitinib restored interdigitation in LR3.2 cells and increased interdigitation in LR1.2 (Fig. 4b). Moreover, EGF treatment increased cell migration in both LR1.2 and LR3.2 (18% and 67%, respectively) (Fig. 4c) indicating that some features of *CASP4*-silencing can be modulated by EGFR signaling and likely by other microenvironmental stimuli. The effect of gefitinib on cell migration in control LR1.2 cells but not in LR3.2 suggests the possibility that autocrine EGF stimulation is partially contributing to cell migration only in LR1.2 cells (Fig. 4c).

### *CASP4*-silencing impairs the *de novo* assembly of adherens junctions

**Figure 5:**
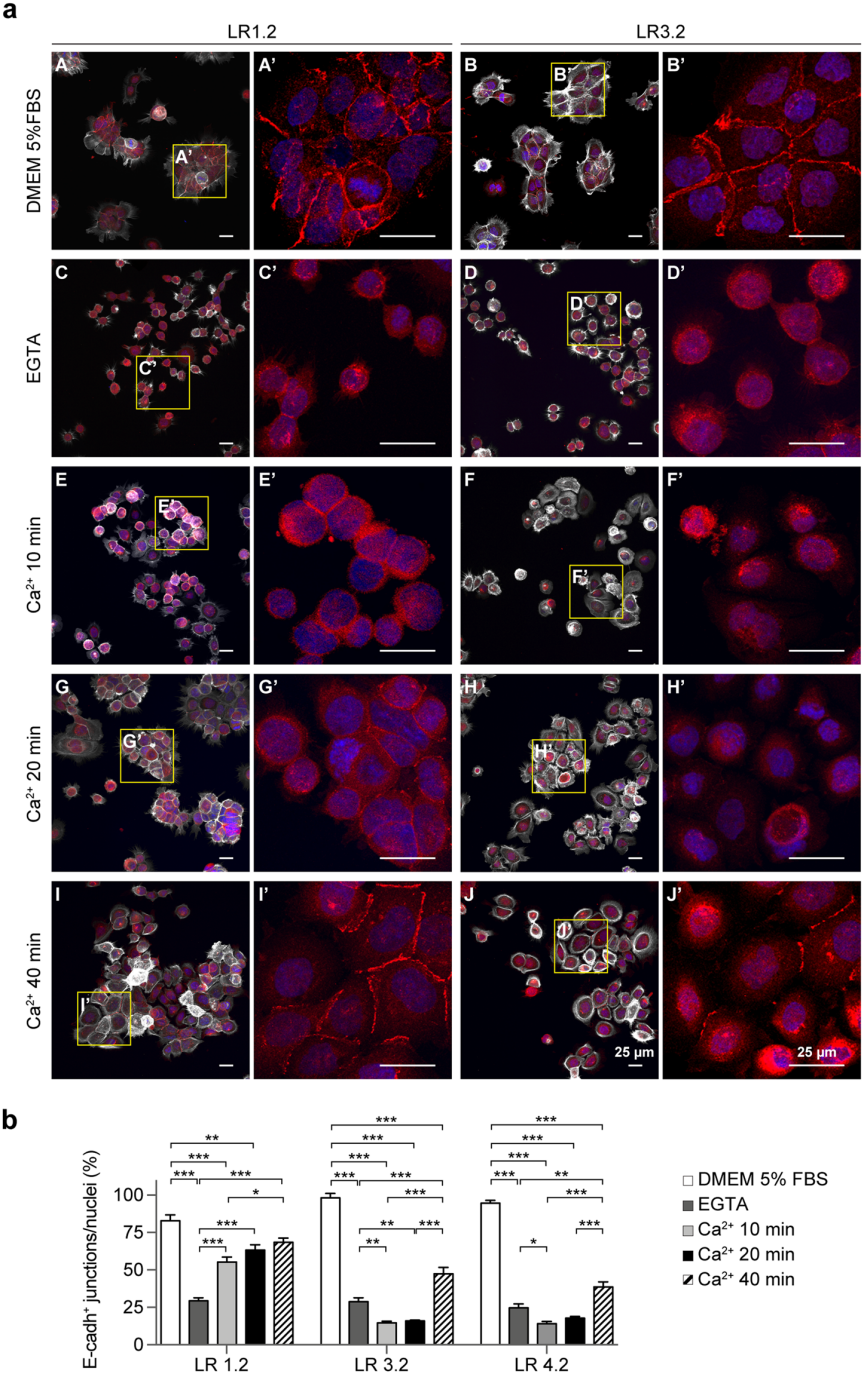
*CASP4*-silencing influences the *de novo* assembly of cell-cell junctions. (**a**) Representative confocal microscopy images of LR1.2 and LR3.2 cells stained with phalloidin-647 (grey) and E-cadherin (red). Cell lines cultured for 16 hours in complete 5% FBS-containing DMEM medium (A,B); cells starved in FBS free-medium and treated with 2 mM EGTA for 1 h (C,D); EGTA treated cells incubated with calcium containing medium for the indicated time points: 10 (E,F), 20 (G,H) and 40 (I,J) minutes. E-cadherin staining (red) of 4x digital enlargements are shown (A’–J’), corresponding to the yellow boxes indicated in A–J. (**b**) Bar plots indicate the percentage of E-cadherin positive mature junctions (length mean 12–45 µm) with respect to total number of nuclei counted by ImageJ in 4–11 fields with a comparable number of cells in two independent experiments (LR1.2: EGTA - DMEM, Ca^2+^ 10 min - DMEM, Ca^2+^ 10 min - EGTA, Ca^2+^ 20 min - EGTA, and Ca^2+^ 40 min - EGTA, p < 0.001; Ca^2+^ 20 min - DMEM, p = 0.009; Ca^2+^ 40 min - Ca^2+^ 10 min, p = 0.02. LR3.2: EGTA - DMEM, Ca^2+^ 10 min - DMEM, Ca^2+^ 20 min - DMEM, Ca^2+^ 40 min - DMEM, Ca^2+^ 40 min - EGTA, Ca^2+^ 40 min - Ca^2+^ 10 min, and Ca^2+^ 40 min - Ca^2+^ 20 min, p < 0.001; Ca^2+^ 10 min - EGTA, p = 0.003; Ca^2+^ 20 min - EGTA, p = 0.009. LR4.2: EGTA - DMEM, Ca^2+^ 10 min - DMEM, Ca^2+^ 20 min - DMEM, Ca^2+^ 40 min - DMEM, Ca^2+^ 40 min - Ca^2+^ 10 min, and Ca^2+^ 40 min - Ca^2+^ 20 min, p < 0.001; Ca^2+^ 10 min - EGTA, p = 0.02; Ca^2+^ 40 min - EGTA, p = 0.002; n = 4–11). Statistical analysis was performed by ANOVA followed by the Tukey’s post-hoc test for every possible pair comparison. Significant p-values are represented by asterisks: *p < 0.05; **p < 0.01; ***p < 0.001. Non-significant p-values are not shown.

To investigate whether CASP4 also plays a role in cell-cell junction assembly, calcium switch experiments were performed. All LR cell lines, plated at 15–20% of confluence, exhibited a smooth pattern of E-cadherin plasma membrane staining at the majority of cell-cell junctions (Fig. 5a). Upon EGTA treatment, E-cadherin staining at the cell-cell junctions strongly decreased in both LR1.2 and LR3.2 cells and upon calcium replacement, AJs were already present at 10 minutes and rapidly increased at 20–40 minutes in LR1.2 cells, whereas in LR3.2 and LR4.2 AJs poorly formed at 10 and 20 minutes and were only partially restored at 40 minutes upon calcium replacement (Fig. 5a and b). The mature cell-cell junctions with a length mean of 12–45 µm were quantified in each cell line upon treatment with EGTA and calcium, at each time point, by confocal microscopy and ImageJ analyses (Fig. 5b).

### *CASP4*-silencing impairs cell detachment and influences number and size of focal adhesions

**Figure 6:**
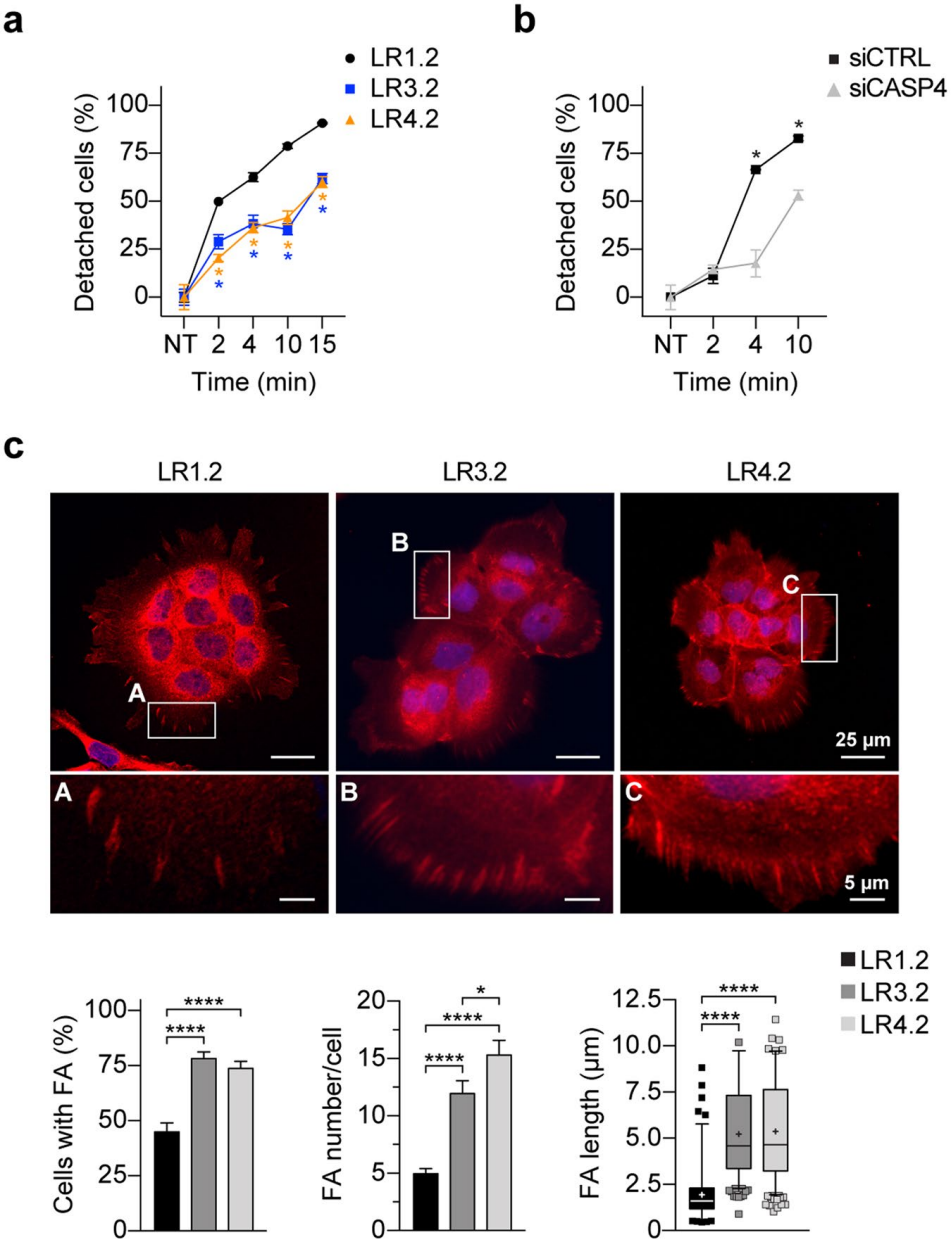
*CASP4*-silencing impairs cell detachment and influences number and size of focal adhesions. (**a,b**) Percentage of cells detached from tissue culture plate upon treatment with trypsin/EDTA is shown in y-axis. The percentage is calculated with respect to untreated LR1.2, LR3.2 and LR4.2 cell lines (**a**) and untreated siCTRL and siCASP4 transfected A431 cells (**b**). Time (min) of incubation in detachment solution is reported in x-axis. Data are representative of three independent experiments (LR1.2 - LR3.2 and LR1.2 - LR4.2 at 2, 4, 10 and 15 min, p = 0.05, n = 3–6; siCASP4 - siCTRL: 4 min, p = 0.04; 10 min, p = 0.05, n = 3). (**c**) Representative confocal microscopy images of talin-stained (red) cells, cultured on poly-L-lysine treated glasses; 4x enlargments are also shown, corresponding to the yellow boxes in LR1.2 (A), LR3.2 (B) and LR4.2 (C). The bar plots indicate the percentage of focal adhesions (FA) positive cells (n = 34–38), the number of FA per cell (n = 39–42) and the FA length (n = 236–597) calculated in clusters of 6–10 cells present in 7–10 fields from three independent experiments (FA positive cells, FA number/cells, FA length: LR1.2 - LR3.2 and LR1.2 - LR4.2, p < 0.0001; FA number/cells: LR3.2 - LR4.2, p = 0.03). Scale bars are indicated. Statistical analysis for every pair-comparison was performed by Wilcoxon rank sum test. Significant p-values are represented by asterisks: *p < 0.05; ****p < 0.0001. Non-significant p-values are not shown.

Focal adhesion complexes regulate the reciprocal communication between cells and the extracellular matrix (ECM) thus mediating cell adhesion. We empirically observed that LR3.2 and LR4.2 cells poorly detach from cell culture monolayer upon trypsin/EDTA treatment. Indeed, while 50% of LR1.2 cells detached very rapidly in approximately 2 minutes and almost all LR1.2 cells detached in 10 minutes, more than 60–65% of LR3.2 or LR4.2 cells were still attached to the dish after 10 minutes (Fig. 6a). Similar resistance to trypsin/EDTA-mediated detachment was also observed in siCASP4 transiently transfected A431 cells (Fig. 6b). Therefore, the number and size of focal adhesions (FAs) were investigated by immunofluorescence and confocal microscopy analysis. LR cells were seeded overnight on poly-L-lysine coated glass round slides in FBS-containing medium at 20% confluence in order to get small groups of 6–10 cells with a clear exposure of cells to ECM (Fig. 6c). The number of cells with talin^+^ FAs and the number of FAs/cell were higher in LR3.2 and LR4.2 cells compared to LR1.2 cells (respectively 74–78% versus 45%; and 1.4- and 2-fold increase respectively) (Fig. 6c). Interestingly FAs in LR3.2 and LR 4.2 cells also showed an increase in FAs length (~1.7 folds), consistently with more mature adhesion features^17^. Similar results were obtained with cells cultured at the same confluence for 16 hours on fibronectin-coated glass slides (Supplementary Fig. S4). Overall, the results on FAs studies while explaining the detachment defects also suggest an increase of cell-matrix interaction in *CASP4*-silenced cells.

### CASP4 promotes lung dissemination of invasive cells

**Figure 7:**
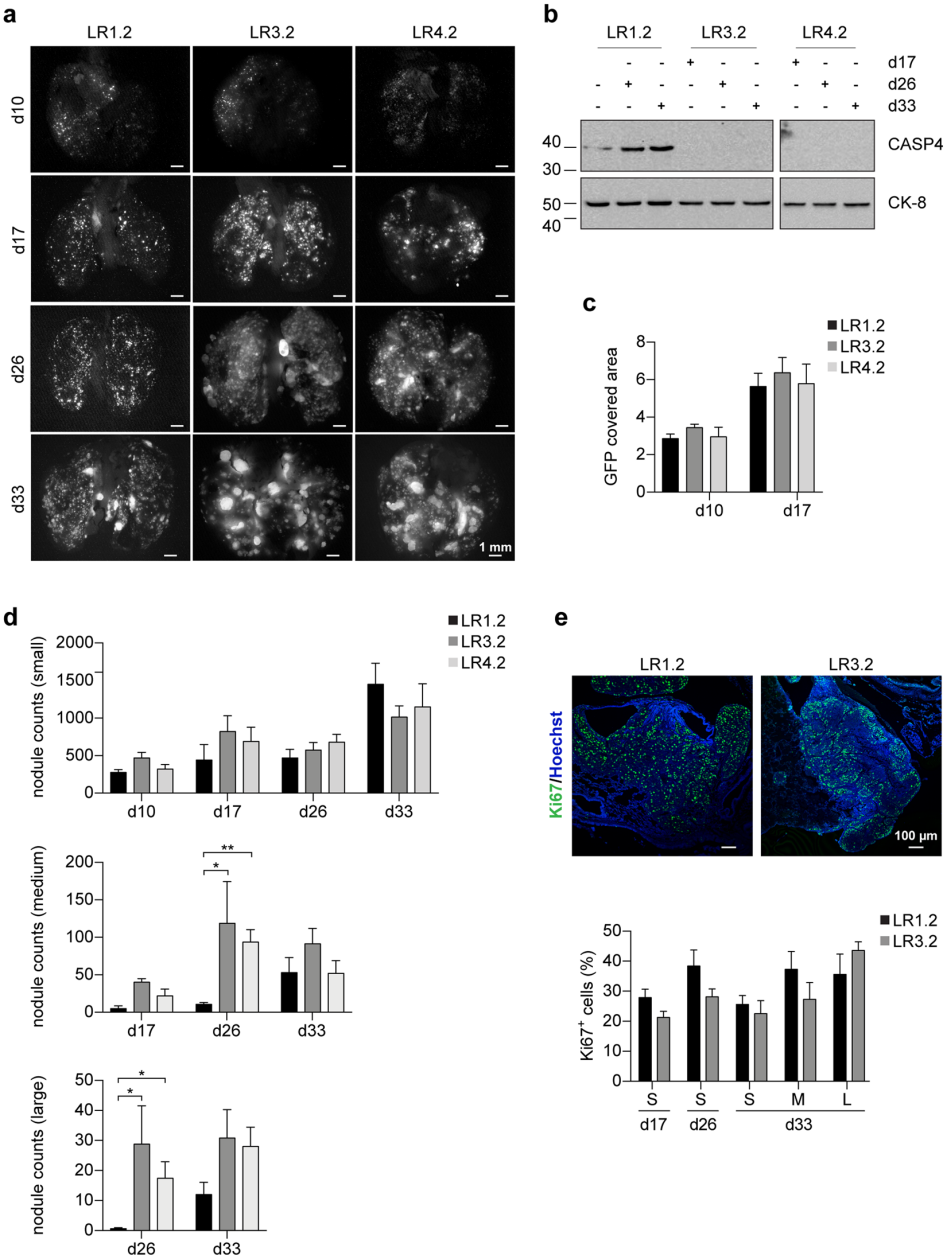
CASP4-silencing influences lung nodules size in nude mice. (**a**) Representative stereomicroscope images of lungs explanted from mice i.v. injected with LR1.2, LR3.2 and LR4.2 cell lines at the indicated time points (day) post-injection (d10, d17, d26, d33). Scale bars = 1 mm. (**b**) Western blot analysis of CASP4 expression on lysates obtained from homogenized lungs explanted from mice i.v. injected with LR1.2, LR3.2 and LR4.2 cell lines at the indicated time points post-injection (d17, d26 and d33). Cytokeratin peptide 8 (CK-8) was used to normalize sample loading. (**c**) Bar plots represent the mean of area covered by GFP at the indicated time points, assessed in three lungs (n = 6, ventral and dorsal lung sides of three mice). (**d**) Bar plots represent the number of small (area < 0.04 mm^2^), medium (0.04 ≤ area ≤ 0.25 mm^2^) and large (area > 0.25 mm^2^) nodules, at the indicated time points, counted by ImageJ in 3–5 lungs/group (day 26 medium nodules: LR1.2 - LR3.2, p = 0.02; LR1.2 - LR4.2, p = 0.008; day 26 large nodules: LR1.2 - LR3.2, p = 0.02; LR1.2 - LR4.2, p = 0.01; n = 3–5). (**e**) Confocal microscopy analysis of Ki67 (green) expression in lung FFPE slide from mice i.v. injected with LR1.2, and LR3.2 cell lines, 33 days upon injection. Images show representative *xy*-plane maximum projection of the specimens; scale bars = 100 µm (Top). Bar plots represent the percentage of Ki67 positive cells in lung nodules (small, n = 10–30; medium, n = 6–7; and large, n = 3–7) of mice i.v. injected with LR1.2 and LR3.2 cell lines. Statistical analysis relative to the comparison of LR3.2 and LR4.2 *CASP4*-silenced cell lines with the control LR1.2 cell line was performed by Wilcoxon rank sum test. Significant p-values are represented by asterisks: *p < 0.05, **p < 0.01. Non-significant p-values are not shown.

In order to investigate the effect of *CASP4*-silencing on tumor invasion and growth, xenograft experiments in athymic nude mice were performed. First, we demonstrated that all LR cell lines can grow subcutaneously in nude mice and are tumorigenic (Supplementary Fig. S5). Next, LR cells invasion in the lung was investigated by i.v. injections. Interestingly, in mice injected with LR3.2 and LR4.2 cells a progressive increase in the size of nodules at day 17-26-33 upon injection was observed (Fig. 7a,d). Instead, in mice injected with LR1.2 control cells, small nodules with a miliaric distribution predominated at all time points following injection (Fig. 7a,d). Next, we excluded that the observed difference was due to diversities in initial lung dissemination and cell homing. Quantification of GFP signal expressed along with the *CASP4* miRNA at day 10–17 upon injection, was similar in all LR lung tissues injected mice (Fig. 7c). In addition, maintenance of *CASP4*-silencing in LR3.2 and LR4.2 lungs was verified by immunoblot (Fig. 7b). The number of Ki67+ cells, taken as a proliferation index, was similar in small nodules at all time points (d17, d26 and d33) and in medium and large nodules at day 33 upon injection, as quantified by IHC analysis of paraffin lung sections of LR cells injected mice (Fig. 7e). The number of medium and large nodules at day 26 upon injection was too small in the lungs of LR1.2 injected mice and therefore a comparative Ki67 expression analysis with LR3.2 was not performed. In conclusion, LR1.2 and LR3.2 cells exhibit comparable Ki67 labeling at all times post-injection in small nodules, which are the vast majority of evolving structures in the lungs, as well as in all classes of nodules at day 33, strongly indicating that the two cell types share the same proliferative rate. Collectively, the data suggest that the large nodules size in the lungs of mice injected with LR3.2 and LR4.2 cells are likely due to defects in cell migration and cell invasion.

## Discussion

The roles of CASP11 and CASP4/5 in the regulation of innate immune responses, in pathogen infections and in immune cells have been well described^18^. In particular, their regulatory role in leucocyte cell migration and pathogen-containing phagosome fusion in macrophages both through actin depolymerization^9,10^. A role in cell migration has also been reported for non-inflammatory caspases. The Drosophila caspase, DRONC, a homologue of mammalian caspase-2 and caspase-9, modulates developmental cell migration due to the interaction of its endogenous inhibitor, Drosophila inhibitor of apoptosis protein 1 (DIAP1), with the GTPase Rac pathway^19^. Caspase-8 gene knockout are early-embryonic lethal in mice; defects in endothelial cell migration have been suggested to play a role in the alteration of the circulatory system of the knockout mutant mice^20^. Interestingly, cell migratory defects also occur in the context of tissue specific caspase-8 knockout and caspase-8 mutant cell lines^21^.

Cell migration is involved in several physiological processes including embryonic development, wound healing, immune events^22,23^ and therefore, defects in cell migration can be linked to various diseases, including cancer progression and metastasis formation. We focused our studies on the consequences of manipulation of *CASP4* expression levels on the behavior of epithelial cancer cell lines. Our results demonstrate that CASP4 is involved in the modulation of adhesion to substrate and cell migration. Overexpression experiments of protease-inactive CASP4.C258S, leading to an increase in cell migration, indicate that caspase activity might not be required as previously reported for CASP11 in lymphocytes^9^. It has been demonstrated that CASP11 interacts with Aip1 and mediates activation of cofilin-mediated actin depolymerization^9^. Although our attempts at co-immunoprecipitating CASP4 and Aip1 in our experimental system failed, the possibility cannot be excluded that this interaction takes place under specific stimuli and mediates cytoskeleton dynamics. In fact, one of the most important properties of cell migration is the ability of cells to fine-tune cytoskeletal structures in response to changing environmental cues such as growth factor stimulation and calcium^24–26^.

Epithelial cells are interconnected through a variety of adhesive structures including E-cadherin containing AJs. The strength and stability of cadherin-dependent adhesion are major determinants of cell migration in epithelial cell sheaths. The extracellular domain of E-cadherin forms Ca^2+^ dependent homophilic trans-dimers, providing specific interaction with adjacent cells, while the cytoplasmic domain is connected to the actin cytoskeleton via anchor proteins called catenins, which in turn are functionally connected to the actin cytoskeleton^27^. The results presented here indicate that, although CASP4 may not be necessary for maintenance of AJs in A431-derived cancer cells, it plays an important role in restoring AJs formation after abrogation of E-cadherin adhesive function. Interestingly CASP4-mediated roles in cell-cell adhesion and cell migration in A431-derived cell lines can also be modulated by EGF stimuli. Indeed, EGF treatment restored cell migration and normalized AJs morphology of *CASP4*-silenced cells. These results raise the interesting possibility that CASP4 can protect the cell-cell barrier during changes in the microenvironment by mild stress conditions such as by pro-inflammatory cytokines or growth factor stimuli that can induce epithelial junction disassembly and increase in epithelial permeability^28^.

Overall our findings suggest that CASP4 can modulate focal adhesion, cell detachment and actin cytoskeletal polymerization, processes that ultimately control cellular migration and cell invasion. The *in vivo* studies in mice show the contribution of CASP4 to tissue invasion and tumor development. Lungs of mice, injected with tumorigenic *CASP4*-silenced cells, were characterized by very large nodules, likely due to impaired cell migration and accumulation of cancer cells unable to invade the surrounding tissue. Importantly because external stimuli (Ca^2+^ and EGF) can modulate the effects of CASP4, the microenvironment can influences CASP4-mediated cellular events including tumor development. Therefore, it is not surprising that in different epithelial tumors the correlation reported for *CASP4* expression levels with the overall survival is varying (https://www.proteinatlas.org). The expression of CASP4 in human cancer samples and its association to patient prognosis has been poorly investigated however some data are already available. Kaplan-Meier curves for CASP4, published on The Human Protein Atlas (https://www.proteinatlas.org/) according to The Cancer Genome Atlas (TCGA) deposited data, show that higher expression of *CASP4* mRNA correlates with a worse overall survival rate in lung (P = 0.02), head and neck (P = 0.03), liver (P = 0.01), pancreatic (P = 0.002), renal (P = 3e-11), and thyroid (P = 0.02) cancers; conversely, in breast (P = 0.01), endometrial (P = 0.02), prostate (P = 0.008) and urothelial (P = 0.004) cancer the worse overall survival is correlated to lower expression of *CASP4*. Moreover, loss of CASP4 expression has been associated with poor prognosis in esophageal squamous cell carcinoma^29^. Interestingly it has been recently developed a sensitive and specific test to quantify CASP4 in plasma and tumor mass. Higher levels of circulating CASP4 was detected in NSCLC patients than in healthy subjects, and higher levels of CASP4 in the tumor mass were associated to reduced overall survival compared to NSCLC patients with lower levels^30^.

## Materials and Methods

### Cell culture and reagents

Human epidermoid A431 cancer cell line, lung carcinoma A549 cell line (ATCC), Phoenix^TM^ Retroviral Packaging Cell Lines Ampho^31^ (ATCC, Manassas, VA, USA) and human kidney 293FT cell line (Thermo Fisher Scientific, Waltham, MA USA) were cultured in Dulbecco’s Modified Eagle’s Medium, 4.5 g/L glucose (DMEM) (BioWhittaker, Lonza, Italy); non small cell lung cancer (NSCLC) HCC827, HCC4006, H1650 and H1975 cell lines, kindly provided by Dr Oreste Segatto, Regina Elena National Cancer Institute, Rome (Italy), and RC2.2 an erlotinib-resistant NSCLC mesenchymal cell line derived from the erlotinib-sensitive HCC4006^32^ were cultured in RPMI 1640 (BioWhittaker, Lonza, USA). Tissue culture media were supplemented with 10 mM Hepes pH 6.98–7.30, 1 mM L-Glutamine, 100 U/ml Penicillin/Streptomycin (BioWhittaker, Lonza,Italy) and heat inactivated 5–10% fetal bovine serum (Sigma-Aldrich). All cells were cultured at 37 °C in a 5% CO_2_ humidified incubator. Complete medium was also supplemented with 0.1 mM MEM non-essential amino acids and 1 mM sodium pyruvate (BioWhittaker, Lonza, Italy) for Hek293FT cell culture. Trypsin/EDTA and Trypsin Neutralizing Solution (TNS) were from BioWhittaker (Lonza, Italy). Human EGF (Sigma-Aldrich) (5 ng/ml) and gefitinib (LC Laboratories) (300 ng/ml) treatments were performed in Opti-MEM reduced serum media (Thermo Fisher Scientific). MTT, 3-(4,5-methylthiazol-2-yl)-2,5-diphenyltetrazolium bromide, poly-L-lysine solution, fibronectin, blasticidin S hydrochloride, puromycin dihydrochloride, polybrene, chloroquine diphosphate salt, protease and phosphatase inhibitors were from Sigma-Aldrich. Antibodies for FLAG (M2), Tubulin (DM1A), Talin (8d4), Cytokeratin peptide 8 (M20), Phalloidin-TRITC, DAPI and Mowiol 4–88 were purchased from Sigma-Aldrich; antibodies for N-cadherin (D4R1H), Vimentin (D21H3) and GAPDH (D16H11) were from Cell Signaling Technology, Danvers, MA, USA; the Caspase-4 (4B9) mouse monoclonal antibody was from Enzo Life Sciences; Hoechst 33258, antibodies for E-cadherin (HECD-1), Ki67 (PA5-19462), Alexa Fluor-488 streptavidin, Alexa Fluor-488, -546 or -647 goat anti-mouse Ig, Alexa Fluor-488 phalloidin and Alexa Fluor-647 phalloidin were from Thermo Fisher Scientific. Biotin-labeled horse anti-rabbit Ig was from Vector Laboratories Burlingame, CA, USA. Goat anti-rabbit IgG (H + L)-HRP and goat anti-mouse IgG (H + L)-HRP were from Bio-Rad.

Plasmid description, siRNA transfection, lentiviral and retroviral infections are described in Supplementary Materials and Methods.

### Calcium influx experiments

LR cell lines were plated at 15–20% of confluence in DMEM 5% FBS-containing complete medium and grown for 16 hours. In order to disrupt E-cadherin-mediated cell-cell adhesion, cells were washed in PBS buffer and treated with EGTA (2 mM) for 1 hour. After extensive washes (10 times) calcium replacement was obtained with DMEM containing 1.8 mM calcium. At the indicated time points (10–40 minutes) cells were washed, fixed in 3.7% paraformaldehyde in PBS and processed for immunofluorescence analysis.

### Cell growth inhibition assays

MTT assay was performed as previously described^32^. Briefly cells (5 × 10^4^ cells/well) in 96-well plate were transfected with siRNA and grown in complete tissue culture medium for 24–72 hours. Next, cells were washed with PBS, incubated for 4 hours with MTT (1 mg/ml) and processed for color detection upon solubilization with DMSO. The samples were quantified spectrophotometrically at 570 nm, with reference wavelength at 630 nm^33,34^.

### Wound-healing assays

A cell-free area was obtained in a monolayer of confluent cells using a sterile yellow tip (time *t*_0_) and then cells were allowed to migrate. Alternatively, the cells were subjected to wound healing assay by using μ-Dish^35 *mm*^ with culture insert with a 500 µm gap following manufacture’s instructions (Ibidi Martinsried, Germany). In experiments with growth factors and inhibitors cells were first starved for 5–6 hours and then stimulated in serum free optimem medium with EGF (50 ng/ml) and/or gefitinib (300 nM) for 48 hours before wound scratch. The monolayer was imaged at different time points (5–24 hours, *t*_*f*_) to monitor wound closure with an Olympus BX41 microscope using a 4–10x lens and an Olympus SP-350 camera. The area of wound coverage was calculated using the ImageJ software^35^. The degree of cell migration was expressed as the percentage of relative wound closure calculated as:

$$\frac{Area\,{t}_{0}-Area\,{t}_{f}}{Area\,{t}_{0}}\,\ast \,100$$

The cells subjected to wound healing assay with the μ-Dish^35 *mm*^ with culture insert were also used for time lapse experiments with Ultraview Vox Spinning Disk Confocal (Perkin Elmer, MA, USA) with a Nikon CS AFC Bino inverted microscope motorized XY-stage (Nikon, Tokjo, Japan), interfaced with Volocity software (Cellular imaging, Perkin Elmer, MA, USA). Briefly, subsequently to the insert removal, the cells were kept in healthy state by using a microscope cage incubator. Time Lapse video-microscopy was carried out with a dry 20x objective. A DPSS solid state laser, operating at 488 nm, was used as excitation source for the EGFP. Next, 4 × 5 fields on the xy stage were selected around the wound area and multiple positions were acquired and automatically tiled, every 15 min for 20 hours.

### Transwell migration assays

Assays were performed using a transwell (Corning Costar, Rochester, NY, USA) containing a polycarbonate membrane filter (8-μm pore size) for 24-well plate according to the manufacturer’s instructions as previously described^36^. Crystal violet staining on the bottom surface of the membrane was eluted in 10% acetic acid; dye reading at an optical density of 590 nm was performed with a Varioskan Lux instrument (Thermo Fisher Scientific).

### Cell lysate preparation and immunoblotting

Cells were lysed in ice-cold RIPA buffer (50 mM Tris-HCl pH 7.5, 150 mM NaCl, 1% NP40, 0.25% sodium deoxycholate, 1 mM EDTA) with protease and phosphatase inhibitors or in SDS-Laemmli sample buffer. Cell extracts (15–30 μg) were then separated by 10% SDS-PAGE and transferred to nitrocellulose membranes 0.45 μm (GE Healthcare Life Sciences). Western blotting was performed according to manufacturer’s instructions. Chemidoc XRS Bio-Rad was used for images acquisition with a chemiluminescent camera.

### Immunofluorescence and confocal microscopy analysis

Cells seeded on poly-L-lysine (100 µg/ml in H_2_O) or fibronectin (2 µg/ml in PBS) pre-coated coverslips or on 35 mm tissue culture plates or in a culture insert μ-Dish^35 *mm*^ plate (Ibidi) were fixed with 3.7% paraformaldehyde in PBS. Cells permeabilized with 0.2% Triton X100 in PBS for 6 minutes at room temperature and treated with blocking solution (1x PBS, 1% BSA) were incubated with primary antibodies, followed by Alexa conjugated secondary antibodies. Nuclei were stained with hoechst 33258 (1 μg/ml) or DAPI (1 μg/ml). Coverslips were finally mounted with Mowiol 4–88 mounting media and viewed on fluorescence Olympus microscope with XM10 camera and processed using CellSens Standard 1.8.1 software. Confocal microscopy analyses were performed with an AX-70a Leica 4d TCS laser scanning confocal microscope or a Fluoview FV1200 Olympus laser scanning confocal microscope. Samples were analyzed using a 63x (NA = 1.4) or 40x (NA = 1.25) oil-immersion lens with optical pinhole at 1 AU. For fluorescence images Argon ion laser at 488 nm, HeNe ion laser at 543 nm and blue diode laser at 405 nm were used as excitation sources. Confocal Z-stacks were collected at 0.4–0.5 μm intervals to a total optical depth of 6–12 μm. Confocal images were processed with Volocity (Improvision, Perkin Elmer), Adobe Photoshop CS4 and ImageJ softwares for image rendering and representation of x/y, x/z or y/z view. All fluorescence image processing steps and cell counts were carried out using the ImageJ software.

### Cytofluorimetric analysis

Transiently transfected A431 cells were collected at the indicated time points and stained with 50 µg/ml of propidium iodide in 0.1%Triton X100/0.1% sodium citrate solution. Acquisition and cell cycle analysis were setup by using a FACSCalibur BD (Becton Dickinson, NJ, USA). Immunocytofluorimetric analysis of LR cell lines stained with anti-FLAG antibody was performed by using the FlowJo software (FLOWJO, LLC Software, USA).

### Cell detachment assays

Cells (1 × 10^5^) were plated in triplicates in 24 wells plate and cultured for 24 hours. Next, detachment was obtained with trypsin/EDTA treatment. Cells still attached to the plate were fixed with 70% ethanol for 10 min and stained with crystal violet (0.1% in distilled water) for 30 minutes. Finally, the dye extracted by incubation of samples in 0.2% Triton X-100 for 5 hours was measured on a Varioskan Lux instrument at an OD of 595 nm. The method was calibrated with a cell-seeding density curve.

### Ethics statement

Animals were subjected to experimental protocols (Authorization N. 81/2013-B and 872/2015-PR) approved by the competent Italian Ministry of Health, DGSF, Roma and experiments were conducted according to the ethical and safety rules and guidelines for the use of animals in biomedical research provided by the relevant Italian law and European Union Directive (Italian Legislative Decree 26/2014 and 2010/63/EU) and the International Guiding Principles for Biomedical Research involving animals (Council for the International Organizations of Medical Sciences, Geneva, CH). All adequate measures were taken to minimize animal pain or discomfort.

### Xenograft in nude mice

Athymic nude female mice (Foxn1^nu^/Foxn1^+^) (Envigo, Italy), 5–10 weeks old, were housed in individually ventilated cages (IVC) under controlled conditions. Subcutaneous tumor xenografts were generated as previously described^32^ with the injection of 0.5 × 10^6^ LR cells into the dorsal flank region. Tumor volume was calculated by caliper measurements of tumor length (L) and width (W) according to the formula: LxW^2^/2. Tumor size and body weight were measured every two days. For the intravenously xenograft, 1–1.2 × 10^6^ LR cells were injected into the tail vein.

### Immunohistochemistry

Immunohistochemistry (IHC) was performed as previously described^32^. Briefly, upon perfusion, excised tumors were formalin fixed paraffin-embedded (FFPE) and slides of 4–8 μm thick were prepared and stained. To analyze nuclear antigens, samples were boiled twice for 20 minutes in antigen retrieval sodium citrate buffer (10 mM pH 6.0) before staining.

### Statistical analysis

The results are shown as mean of experimental replicates with standard error as variability measure. Shapiro-Wilk test and Bartlett’s test were performed to check the assumption of normality of datasets distributions and homoscedasticity, respectively. For normally distributed data we used two sample t-test for the comparison of two groups, and ANOVA with subsequent Tukey’s post-hoc test for multiple comparisons. Dataset with a non-normal distribution or with a small sample size (n < 10) were analysed by using Wilcoxon rank sum test. P-values lower than 0.05 were considered significant. Statistical analysis and graphs were performed using R (version 3.4.3) and GraphPad Prism software version 7 (GraphPad Software, CA).

## Electronic supplementary material

Supplementary Information

Supplementary movie1
