# Supplementary material for: CASP4 gene silencing in epithelial cancer cells leads to impairment of cell migration, cell-matrix adhesion and tissue invasion
Source: Sci Rep. 2018 Dec 7;8:17705. doi: 10.1038/s41598-018-35792-8 (PMC6286322; doi:10.1038/s41598-018-35792-8)

CASP4 gene silencing in epithelial cancer cells leads to impairment of cell migration, cell-matrix adhesion and tissue invasion

Giuliana Papoff[1*], Dario Presutti[1], Cristiana Lalli[1], Giulia Bolasco[2], Simonetta Santini[1], Candida Manelfi[1], Valentina Fustaino[1], Stefano Alemà[1] and Giovina Ruberti[1*]

[1] National Research Council, Institute of Cell Biology and Neurobiology - Campus Adriano Buzzati-Traverso Via E. Ramarini, 32 00015 - Monterotondo (Rome) - Italy

[2] EMBL Epigenetic and Neurobiology Unit - Rome – Italy

*Correspondence to:

giuliana.papoff@cnr.it

giovina.ruberti@cnr.it

Supplementary - Materials and Methods

Plasmids. pLenti6.4/promoter/MSGW/EmGFP-miRNA expression plasmids, coding for miRNA targeting CASP4 in frame with GFP, were generated by using the BLOCK-iT^{TM} HiPerform Lentiviral Pol II miR RNAi Expression System with EmGFP (Thermo Fisher Scientific). In particular miRNACASP4_Hmi402233, targeting nucleotides 305-325 of CASP4 coding sequence and miRNA CASP4_Hmi402234 targeting nucleotides 480-500 were designed according to manufacturer's instructions. The pcDNA6.2-GW/EmGFP-miR-neg scrambled expression vector was used to generate the pLenti6.4/promoter/MSGW/EmGFP-miRNA scramble expression vector.
pFLAG-CMV2_CASP4 expression vector was generated by RT-PCR from peripheral blood lymphocytes mRNA with the GR226 5'-CCCGGTCGACGCAGAAGGCAACCACAGA-3' and GR253 5'-CCCGGGATCCTCAATTGCCAGGAAAGAGG-3' oligonucleotides. Next CASP4 insert was subcloned in the Bam HI site of pBABEpuro expression vector (Addgene), generating FLAG-CASP4 expression vector. pFLAG-CMV_CASP4.C258S expression vector was generated by site directed mutagenesis of pFLAG-CMV2_CASP4 with the oligonucleotides: GR 350 5'-GTCATCATTGTCCAGGCCAGCAGAGGTGCAAACCGTGGG-3' and GR 351 5'-CCCACGGTTTGCACCTCTGCTGGCCTGGACAATGATGAC-3'. Next CASP4 insert was subcloned in the Bam HI site of pBABEpuro expression vector (Addgene), generating FLAG-CASP4.C258S expression vector. All expression vectors were subjected to nucleotide sequencing. pkat2 retroviral packaging[1] was gently provided by Dr. Maurizia Caruso, IBCN-CNR, Italy.

siRNA trasfections. A431, A549, HCC827, H1650, H1975 and HCC4006 cell lines were transfected with D-004404-01-0020 (Dharmacon), siRNA#1 (GGACUAUAGUGUAGAUGUA), siRNA#2 (AAGUGGCCUCUUCACAGUCAU), siRNA#3 (AAGAUUUCCUCACUGGUGUUU), and siLuciferase Invitrogen siRNA#4 (CGUACGCGGAAUACUUCGATT) (Thermo Fisher Scientific) by lipofectamine RNAiMax, according essentially to manufacturer's instructions. Briefly, cells were plated in optimem glutamax medium at approximately 2-5 x 10^5 cells in 35 mm plates or 2-5 x 10^4 cells in 96 wells plates and transfected 24 hours upon plating with siRNA (100-200 nm). Next, 72 hours later if not otherwise indicated, cells were collected and lysed for western blotting or

processed for MTT assays, cytofluorimetric analysis, wound healing assays or cell detachment experiments.

Lentiviral infection. 293FT cells were transfected with pLenti6.4/promoter/MSGW/EmGFP-miRNA expression plasmids premixed with the ViraPower packaging mix by using lipofectamine 2000 reagent (Thermo Fisher Scientific). Supernatants were collected 72 hours later and frozen or immediately used to infect A431 cell line, at 1:10 dilution in DMEM 10% FBS, with 6 µg/ml of polybrene. At 24 hours post-infection, the virus containing medium was removed and the cells subjected to a second infection cycle. Finally, blasticidin was added 24 hours later for selection of positive LR clones, isolated in approximately 15 days. Gene silencing *of CASP4* in LR3.2 and LR 4.2 cell lines was stable under blasticidin selection after 10-12 passages.

Retroviral infection. Phoenix ampho packaging cells (1.5-2 x 10^6) were plated in 60 mm plates in DMEM complete medium without antibiotics in 5% FBS 24 hours before transfection. Cells at 70-80% confluence were treated for 5 min with chloroquine before infection with pBABEpuro expression vectors (2.5 µg) and pkat2 (0.6 µg) plasmid in optimem glutamax with lipofectamine 2000 (Thermo Fisher Scientific). Next, 6-8 hours post-infection medium was replaced with fresh DMEM complete medium. Supernatant was collected after 48 hours, centrifuged and 0.45 µm filtered. A431 cells were infected with retroviral supernant (1:2 dilution) by using 5 µg/ml polybrene and selected with puromycin (1 µg/ml) for approximately 15 days.

Supplementary - Figure Legends

Supplementary Fig. S1. ***CASP4*-silencing in A431 cells impairs cell migration but not cell growth or apoptosis**. (**a**) Analysis of cell migration by wound healing assays of A431 transfected with the *CASP4* siRNA #2 and #3. siCTRL was luciferase siRNA. The wound closure was quantified at 8 hours post-wound by measuring the cell-free area using the ImageJ software. Bar plots represent the percentage of relative wound closure calculated as described in materials and methods. Data were obtained by the analysis of 5-9 images (#2: p=0.0002, n=7-9; #3: p=0.008, n=5). (**b**) Analysis of MTT survival assays of siCTRL (luciferase) and siCASP4 A431 cells at 24, 48 and 72 hours upon transfection. Data points represent the optical reading ($OD_{570\ nm}$) values of three independent experiments (n=9). (**c**) Cell cycle analysis of siCTRL and siCASP4 transfected A431 cells by cytofluorimetric acquisition of propidium iodide stained cells. The percentage of hypoploid cells is indicated. Statistical analysis was performed by Wilcoxon rank sum test for the comparison of siCASP4 with siCTRL transfected A431 cells. Significant p-values are represented by asterisks: ** p<0.01; *** p<0.001. Non-significant p-values are not shown.

Supplementary Fig. S2. Western blot analysis of mesenchymal markers in A431 silenced cells. (**a**) Western blot analysis of CASP4 and tubulin expression in A431 cells transfected with the indicated siRNA (200 nM). (**b**) Western blot analysis of N-cadherin, E-cadherin, Vimentin and GAPDH expression in A431 cells transfected with the indicated siRNA (200 nM) in two independent experiments. RC2.2 was used as positive control. It is a NSCLC erlotinib-resistant cell line derived in the laboratory from the erlotinib-sensitive HCC4006 cell line with a mesenchymal phenotype, previously described[2].

Supplementary Movie 1. LR cell migration

LR1.2 and LR4.2 cell lines were subjected to time-lapse video-microscopy in wound healing assay. Mosaics of 4x5 fields were recorded at the spinning disk (Ultraview Vox Spinning Disk Confocal, Perkin Elmer) for 20 hours, taking pictures every 15 minutes.

Supplementary Fig. S3. Infection of LR1.2 cell line with FLAG-CASP4 retroviral supernatant. (**a**) Cytofluorimetric analysis of LR1.2 cells infected with retroviral particles driving FLAG-CASP4 or FLAG-CASP4.C258S cDNA, stained with FLAG antibody. The percentage of FLAG positive cells is indicated. Empty vector corresponds to pBABEpuro supernatant infected cells. (**b**) Representative confocal microscopy images of LR1.2_FLAG-

CASP4 wounded areas, stained with FLAG reactive antibody (red) and hoechst (blue); the wounded area is included in white dashed line. Scale bar = 25 μm.

Supplementary Fig. S4. *CASP4*-silencing influences number and size of focal adhesions on fibronectin. Bar plots indicate the percentage of focal adhesions (FA) positive cells (n=16), the number of FA per cell (n=39-58) and the FA length (n=227-242) counted in clusters of 6-10 cells present in 15 fields from two independent experiments (FA positive cells, FA number/cells, FA length: LR1.2 - LR3.2 and LR1.2 - LR4.2, $p<0.0001$). Statistical analysis for every pair-comparison was performed using Wilcoxon rank sum test. Significant p-values are represented by asterisks: **** $p<0.0001$. Non-significant p-values are not shown.

Supplementary Fig. S5. A431 and LR-derived cells are tumorigenic in nude mice. Representative stereomicroscope images in fluorescence (FL) or in bright-field (BF) of tumor nodes obtained in athymic nude mice subcutaneously injected with the indicated cells. Scale bars = 2 mm. Tumor growth curves of subcutaneous xenograft nodules (n=3-8) are shown in the plot. Statistical analysis was performed by Wilcoxon rank sum test at the last time point (day 26) for every pair-comparison and resulted in not significant p-values.

Supplementary Fig. S6. Full-length/uncropped immunoblots

Supplementary - References

1. Finer, M. H., Dull, T. J., Qin, L., Farson, D. & Roberts, M. R. kat: a high-efficiency retroviral transduction system for primary human T lymphocytes. *Blood* **83**, 43-50, (1994).
2. Presutti, D. *et al.* MET Gene Amplification and MET Receptor Activation Are Not Sufficient to Predict Efficacy of Combined MET and EGFR Inhibitors in EGFR TKI-Resistant NSCLC Cells. *PLoS One* **10**, e0143333, https://doi.org/10.1371/journal.pone.0143333 (2015).

Fig. S1

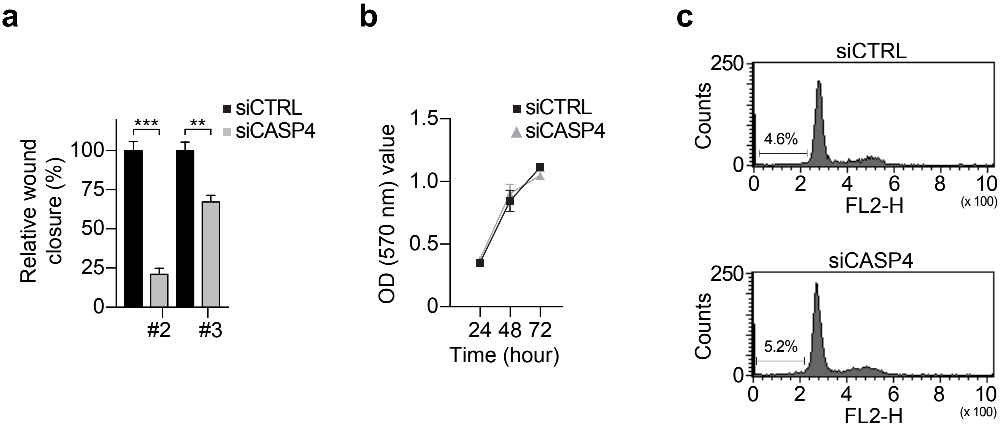
a
siCTRL
siCASP4
***
**
Relative wound closure (%)
100
75
50
25
0
#2
#3
b
siCTRL
siCASP4
OD (570 nm) value
1.5
1.0
0.5
0
24 48 72
Time (hour)
c
siCTRL
250
Counts
4.6%
0
0 2 4 6 8 10
FL2-H
(x 100)
siCASP4
250
Counts
5.2%
0
0 2 4 6 8 10
FL2-H
(x 100)

Fig. S2

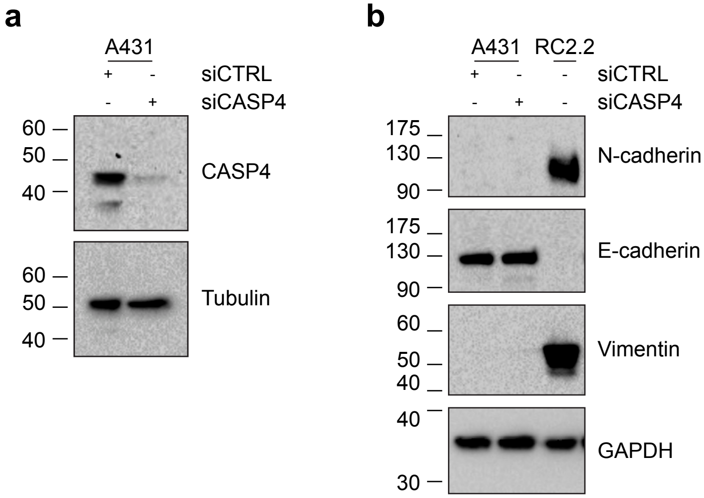
a
A431
+ - siCTRL
- + siCASP4
60
50
40
CASP4
60
50
40
Tubulin
b
A431 RC2.2
+ - - siCTRL
- + - siCASP4
175
130
90
N-cadherin
175
130
90
E-cadherin
60
50
40
Vimentin
40
30
GAPDH

Fig. S3

a

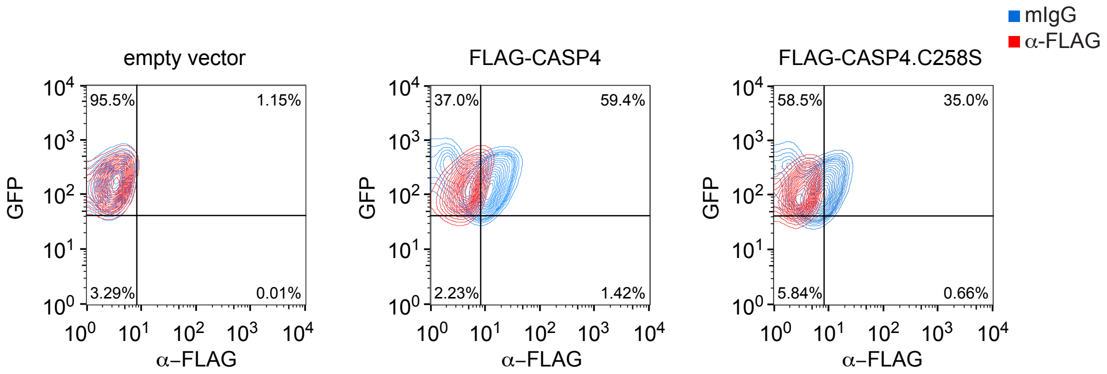
mIgG
α-FLAG
empty vector
FLAG-CASP4
FLAG-CASP4.C258S
95.5%
1.15%
3.29%
0.01%
37.0%
59.4%
2.23%
1.42%
58.5%
35.0%
5.84%
0.66%
GFP
α–FLAG
10^0
10^1
10^2
10^3
10^4

b

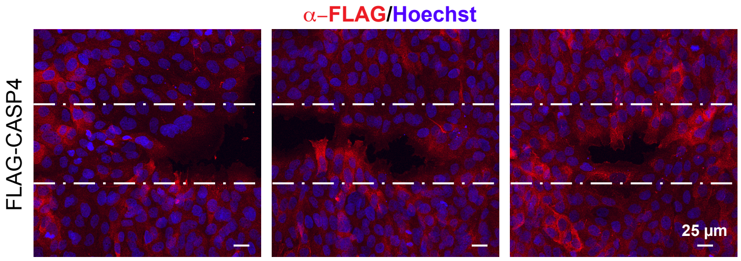
α–FLAG/Hoechst
FLAG-CASP4
25 μm

Fig. S4

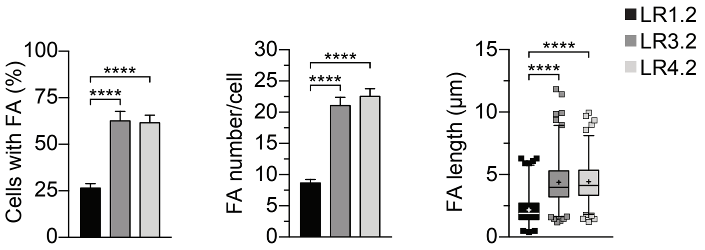
LR1.2
LR3.2
LR4.2
Cells with FA (%)
100
75
50
25
0
****
****
FA number/cell
30
25
20
15
10
5
0
****
****
FA length (µm)
15
10
5
0
****
****

Fig. S5

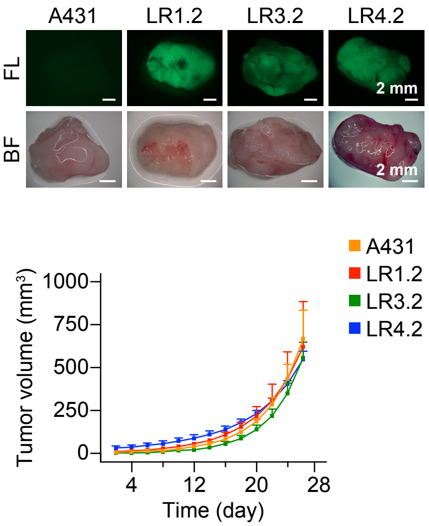

A431
LR1.2
LR3.2
LR4.2
FL
BF
2 mm
2 mm
A431
LR1.2
LR3.2
LR4.2
Tumor volume (mm^3)
1000
750
500
250
0
4
12
20
28
Time (day)

Fig. S6

Figure 1a

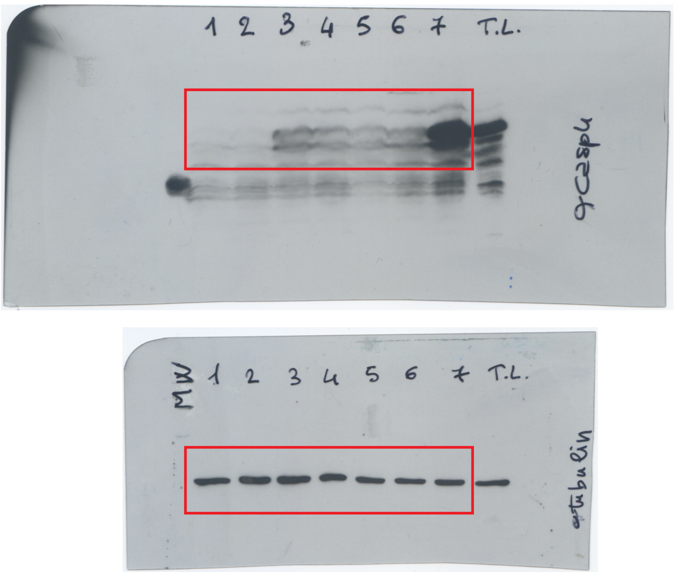

Figure 1d

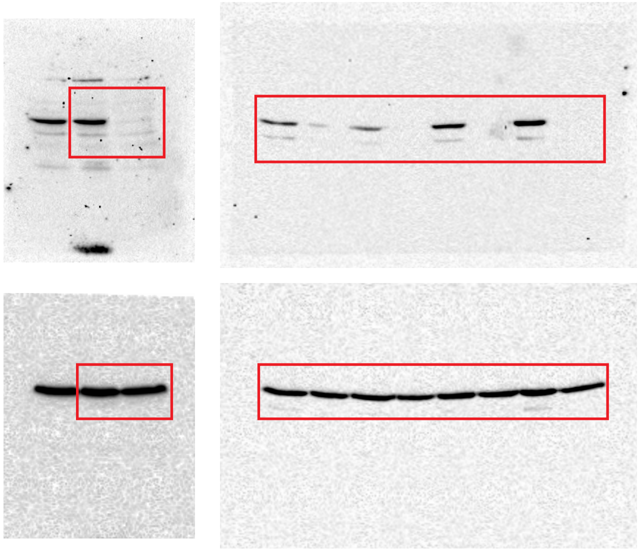

Figure 3a

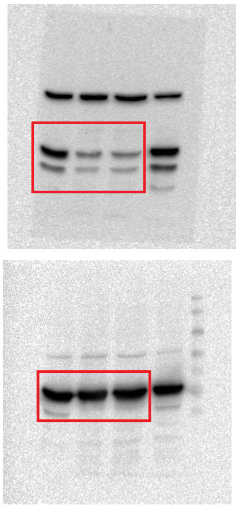

Figure 7b

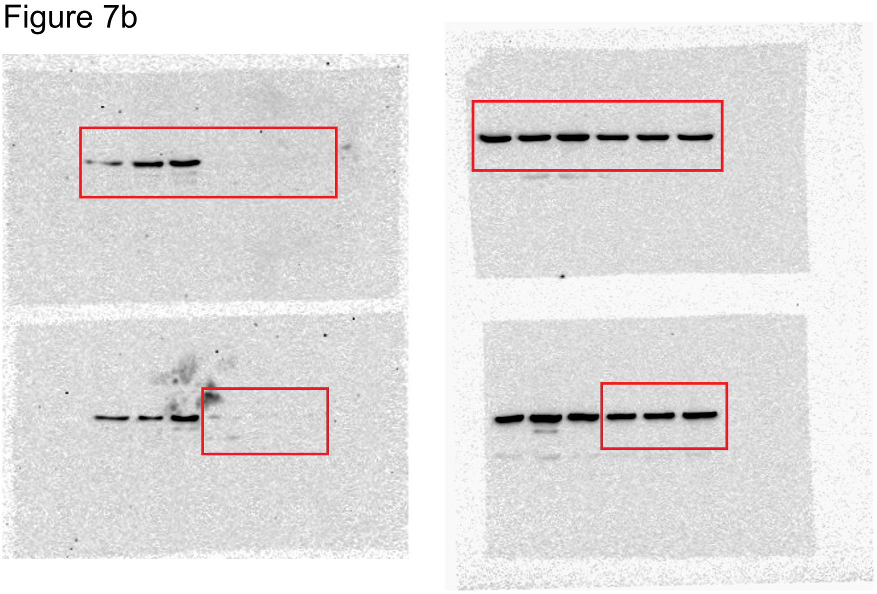

Figure S2a

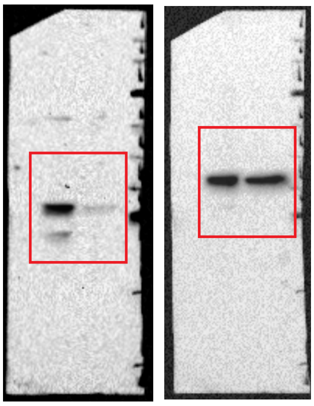

Figure S2b

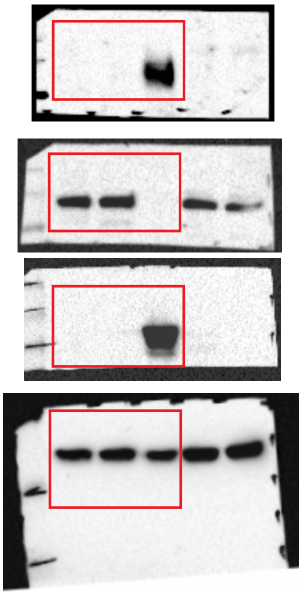

Supplement: Supplementary file 1 — Supplementary Information [file 41598_2018_35792_MOESM1_ESM.pdf]
